# Saccades track visual associative memory processes with precision and sensitivity

**DOI:** 10.1093/braincomms/fcaf219

**Published:** 2025-06-04

**Authors:** Simon Henin, Eden Tefera, Helen Borges, Orrin Devinsky, Charan Ranganath, Anli Liu

**Affiliations:** Comprehensive Epilepsy Center, NYU School of Medicine, NYU Langone Health, New York, NY 10016, USA; Comprehensive Epilepsy Center, NYU School of Medicine, NYU Langone Health, New York, NY 10016, USA; Comprehensive Epilepsy Center, NYU School of Medicine, NYU Langone Health, New York, NY 10016, USA; Comprehensive Epilepsy Center, NYU School of Medicine, NYU Langone Health, New York, NY 10016, USA; Department of Psychology, University of California, Davis, CA 95616, USA; Comprehensive Epilepsy Center, NYU School of Medicine, NYU Langone Health, New York, NY 10016, USA

**Keywords:** eye tracking, temporal lobe epilepsy, memory

## Abstract

Humans primarily use vision to engage with and learn about the world. The hippocampus plays a crucial role in binding visual experiences of people, objects and contexts over time to create event memories. Thus, eye tracking could read out hippocampal dynamics in a precise and sensitive manner. Furthermore, eye tracking could potentially detect subjective memory decline reported by temporal lobe epilepsy patients that is missed by standardized cognitive testing. We asked whether eye movements could precisely and sensitively detect memory variability within trials and between subject cohorts. We predicted that (i) eye-tracking behaviour during visual retrieval could be validated against accuracy-based tests and that (ii) memory failures would be characterized by distinct spatiotemporal patterns of visual scanning. Fourteen healthy controls and 30 temporal lobe epilepsy patients participated in a visual object association task while eye movements and pupil size were recorded. We found a difference in accuracy during retrieval between healthy controls and temporal lobe epilepsy patients. Correct retrieval trials correlated with fewer saccades, early target preference, and a more organized search pattern. Eye-movement patterns could predict retrieval accuracy at the single trial level with outstanding performance, with percentage of gaze time on the target versus the lure as the most important features. Even during correct retrieval trials, temporal lobe epilepsy patients exhibited a more chaotic scanning pattern compared to healthy controls, suggesting a weaker memory trace. Healthy versus epilepsy diagnosis could be predicted with good performance, with trial entropy and pupillary changes as key predictive factors. Saccade patterns correlated with individual subjects’ accuracy scores and performance on standardized cognitive tests but provided a greater range of performance. In summary, scanning behaviour provides a continuous measure of associative memory function that capture meaningful variability during trials, between trials, and between subjects. Thus, eye tracking could be a precise and sensitive method to detect subtle memory decline in temporal lobe epilepsy or other neuropsychiatric populations with memory impairment and may generate precise behavioural phenotyping in research settings.

## Teaser

Eye tracking can yield quantitative, real-time measurements of memory behaviour. Accurate visual association is characterized by fewer saccades, early target preference, and an organized scanning pattern. Even during correct retrievals, patients with temporal lobe epilepsy demonstrate a less efficient visual search compared to healthy controls, suggesting a weaker memory trace.

## Graphical Abstract

**Graphical Abstract:**
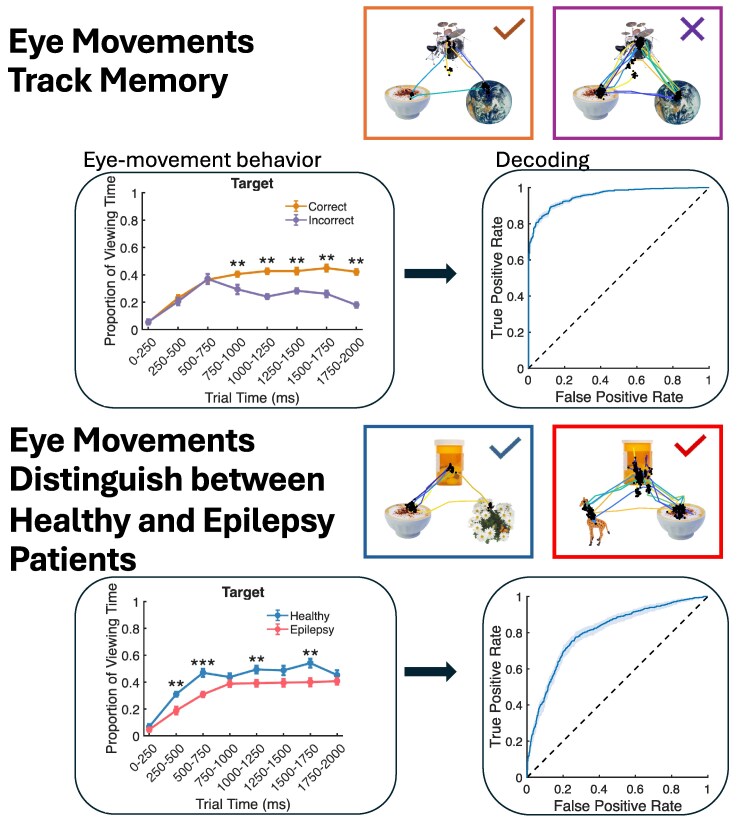

## Introduction

While exploration in rodents mainly involves olfaction, locomotion, and head movements,^1^ humans primarily depend on vision to scan and extract information about the environment. Oculomotor activity is the behavioural expression of visual exploration and recognition in primates,^2^ analogous to rodent navigation. Because scanning occurs within tens to hundreds of milliseconds, eye tracking could track hippocampal function in real time as humans construct and remember experiences.^3,4^

Epilepsy patients commonly report memory difficulty, but traditional neuropsychological testing often misses subtle dysfunction. Typical neuropsychological testing performed throughout a 30-min delay can miss subjective memory impairment in temporal lobe epilepsy (TLE) patients, whose deficits may be apparent only after days or weeks.^5,6^ Clinical contributions to the degree of cognitive impairment include early onset of epilepsy, frequency of seizures and interictal epileptiform discharges, low education level, and polypharmacy.^7,8^ Pathophysiologically, TLE patients have selective vulnerability of the dentate gyrus and subiculum, followed by CA4, CA1, CA2, and CA3 subfields.^9,10^

When scanning the environment, eye movements rapidly switch between saccades and fixations. ‘Saccades’ are sudden ballistic eye movements between objects or features in the environment. ‘Fixations’ involve prolonged gaze on attended objects. Eye movements are not random, but influenced by (i) stimulus features (e.g. colour, luminosity) and (ii) episodic and semantic memories.^11^ Monkeys, human infants and healthy adults prefer looking at novel versus familiar objects^12-15^ More gaze fixations occur when viewing new or manipulated portions of a particular scene, compared to familiar scenes, even if the subject is unaware of the manipulation.^16-18^ Eye movements reveal relational memory^2,11,19,20^ and temporal sequences.^21^ Spontaneous eye movements during exploration of a scene are re-enacted during second viewing the original picture.^22,23^ In contrast to healthy subjects, patients with hippocampal damage have impaired novelty preference, revealed by equal time looking at new and old objects.^19,24,25^ Hippocampal damage reduces spontaneous oculomotor exploration, scene discrimination, perception of spatial relationships^26-29^ and spatial and contextual memory.^30^ While there is strong evidence that memory-guided visual scanning is hippocampally dependent, whether eye movements can detect subtle differences in hippocampal function across trials and between individuals is unclear.

We asked whether eye-movement patterns could be (i) precise, as validated by trial accuracy, comparison to Montreal Cognitive Assessment (MOCA) scores, and subject diagnosis; and (ii) sensitive, as measured by differences in behaviour within and between single trials. We studied the relationship of eye movements to explicit memory performance in 14 healthy subjects and 30 TLE patients. We used a visual inference task^31^ that requires subjects to retrieve (i) object pairs that were learned at the same time and context (direct associations) and (ii) object pairs that were presented at different times and contexts (indirect associations)—similar to what our brain must do in everyday life.^18,32,33^ Remembering direct associations (e.g. Mary and Joe work together, Joe and Frank work together) and the ability to infer new relationships (e.g. Mary and Frank work together) are foundational to episodic memory and dependent on hippocampal function.^31^

We hypothesized that eye-movement patterns could precisely and sensitively measure associative memory function by (i) decoding retrieval accuracy, (ii) capturing the variance in memory performance within and between HCs and TLE patients as confirmed by clinical neuropsychological testing (MOCA), and (iii) distinguishing between successful and failed retrieval processes in a time-resolved manner. We discovered that optimal retrieval performance was characterized by fewer saccades, early preference for the target object and a more succinct, organized search pattern. In contrast, incorrect retrieval trials were associated with more chaotic and prolonged visual scanning. Disorganized visual scanning was observed more often in TLE patients, even during correct trials, suggesting a weakened memory trace. These key eye-tracking features could predict explicit accuracy of retrieval with outstanding performance [area under the curve (AUC) 0.9437] and subject diagnosis with very good performance (AUC 0.8017). Scanning behaviour on this visual inference task strongly correlated with performance on a standardized cognitive screen (MOCA). Overall, eye tracking can provide objective, precise, sensitive and real-time measurements of associative memory function, without requiring explicit feedback. Visual scanning behaviour during a visual association task could be useful to detect subtle memory decline in memory function in TLE patients and other patients with hippocampal dysfunction.

## Materials and methods

### Subjects

This study was conducted following protocols approved by the New York University Institutional Review Board. All study activities complied with regulations for human subject research, and all data were collected during a single study session. We recruited TLE patients and healthy controls (HCs) at the NYU Comprehensive Epilepsy Center from 2018 to 2023. HCs were included if they were between the ages of 18 and 60 years, did not have a self-reported history of neurological or psychiatric disease and earned a normal score on the MOCA (≥26/30). The MOCA is a widely used cognitive screening tool assessing multiple cognitive domains, including memory, attention, executive function, visuospatial construction, naming and orientation. Patients scoring ≥ 22/30 on the MOCA were included. A lower threshold for TLE patients was chosen to include patients with mildmemory impairment. Epilepsy localization was determined by seizure semiology, MRI brain and EEG concordance, and adjudicated by a board-certified neurologist and epileptologist (A.L.). Only patients with probable or definite unilateral temporal lobe epilepsy were included (meaning at least two concordant diagnostic criteria without discordant criteria).

### Visual association task

**Figure 1:**
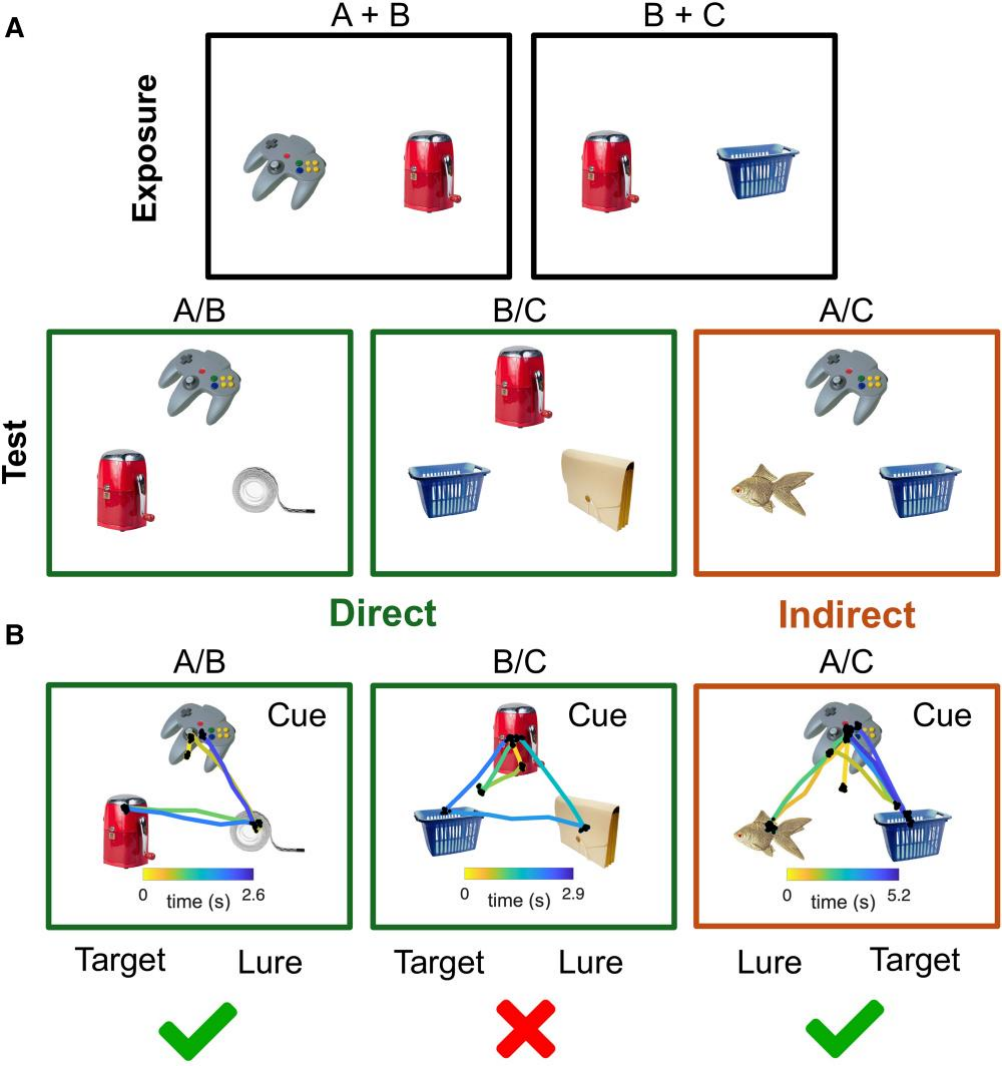
**Experimental design.** (**A**) Top row: during the exposure phase, subjects were shown 60 pairs of objects (A + B, B + C) for 1 s. Bottom row: during the test phase, subjects were shown triads, with the cue object at the top and two choices on the bottom, the correctly paired object (‘target’) and another object in the set (‘lure’). There were 60 trials probing direct associations (A + B, B + C), and 30 trials probing indirect associations (A + C). All objects were shown during the exposure phase. Subjects were given unlimited time during retrieval. Eye movements were recorded during the entire session. (**B**) Example of eye-tracking behaviour responses during correct direct association between ‘cue and target’ (A/B), incorrect direct association between ‘cue’ and ‘lure’ (B/C) and correct indirect association between ‘cue’ and ‘target’ (A/C). All object images were made freely available from https://bradylab.ucsd.edu/stimuli.html.^34^

We developed a visual object association task based on a previously published paradigm (Fig. 1).^31^ After an exposure block, subjects were given two testing blocks (direct association and indirect association). During the exposure phase of each block, 60 pairs of objects were selected from a standardized object database.^34^ The exposure phase for each pair lasted 1 s. Objects were selected for similar size, resolution and luminance. Some pairs included two new objects shown side by side (A + B), and some pairs included one familiar object and one novel object (B + C) (Fig. 1A). All objects shown in pairs during the encoding period (60 objects) were shown again during the retrieval period, which consisted of two retrieval blocks of direct associations (2 × 30 trials) and one block of indirect associations (1 × 30 trials). Thus, all objects presented during the test phase were familiar objects. During the 60 direct association retrieval trials, subjects were probed for memory of object pairs shown at the same time during the first block (A + B or B + C) and used the mouse to point and click on the correctly paired object. During the 30 indirect association testing trials, subjects were probed for the memory of object pairs shown at different times, thus requiring the subject to make a novel association between objects shown at different times (e.g. encoding: A + B, B + C, retrieval: A + C). In every test trial, subjects were shown a cue object on the top row and two potentially associated objects on the bottom row (‘target’ and ‘lure’) (Fig. 1B). Subjects were not time-restricted during the test phase. Each retrieval trial ended when the subject chose the target or lure with a mouse click. Accuracy of retrieval was defined at the single trial level (correct vs. incorrect) and by the percentage of objects (targets) correctly associated with the anchor object (cue) over the entire session. Chance performance would be 50%.

### Eye tracking and analysis

**Figure 2:**
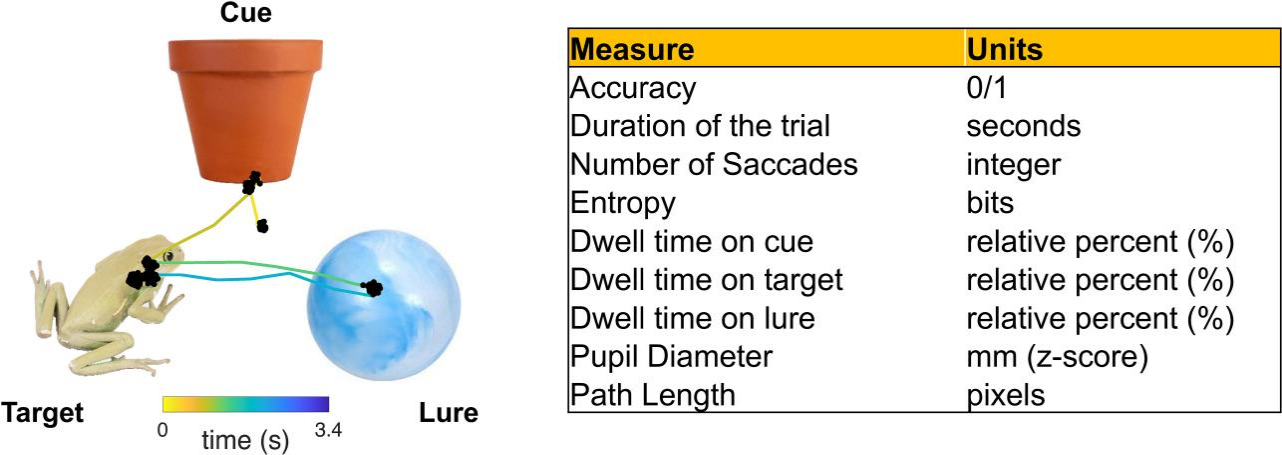
**Eye-tracking overview.** Example eye movements during a visual association task and eye-tracking metrics.

Eye movements and pupillometry were recorded using portable Tobii eye-tracking systems (Model 4c, sampling rate 90 Hz; Tobii Fusion Pro sampling rate of 120 Hz). Eye trackers were placed at the bottom of the laptop screen, with the subject’s viewing distance ∼64 cm from the screen. For each subject, the eye tracker was calibrated using five-point calibration with different fixation locations around the screen. Calibration errors across subjects averaged 1.16 ± 1.4 degrees of visual angle (d.v.a.), with no difference in calibration accuracy between HC and TLE groups [HC: 1.03 ± 0.97 d.v.a., TLE: 1.23 ± 1.23 d.v.a., *t*(42) = 0.4712, *P* = 0.64]. When the subject shifted in position, recalibration of the eye tracker was performed by a member of the study team to maintain high-quality recordings. Eye movements were analysed offline and saccades and fixations were extracted using an automated procedure.^35^ Approximately 8% of trials included more than 30% invalid data points and were excluded from further analysis. Eye movements were extracted from the eye-tracking data during the exposure and test phases of the study (Fig. 2, see Supplementary Table 1 for a full list of eye-tracking features extracted). For each trial, basic eye-tracking metrics (e.g. number of saccades, number of fixations, dwell time on each object) were computed and tabulated for each subject. Entropy was calculated for each trial by generating a two-dimensional grayscale heat map of the participant’s eye gaze positions during the trial and computing visual entropy (e.g. Shannon’s entropy).

Pupil size acquired from the eye tracker (in mm) was *z*-scored across each participant’s session to provide normalized values of pupil changes.^36^ We selected this approach because pre-saccade pupil diameter is influenced by the prior saccade’s pupillary response. Changes in pupil size were further analysed by time-locking to saccade initiation and fixation onset for both correct and incorrect trials. Because all objects and their combinations were presented in the same way to every subject, our comparison of pupil size between correct and incorrect conditions accounts for any low-level visual features (e.g. luminance or object complexity) which could influence pupil size fluctuation.

### Statistical analysis

All comparisons of eye-tracking metrics (e.g. trial duration, no. of saccades) observed between conditions (e.g. correct versus incorrect, TLE versus HC subjects) were assessed using a non-parametric Wilcoxon’s rank sum test. To analyse differences in viewing times (e.g. dwell time to target) across time, the average dwell ratios for each object (cur, target, lure) were binned in 250 ms windows and averaged per participant. Subsequent binned ratios were analysed between conditions (correct versus incorrect) or subject group (healthy versus TLE) using a Wilcoxon’s rank sum test and corrected for multiple comparisons using the false-discovery rate procedure. Statistical significance of any differences in pupil size between conditions were assessed via non-parametric cluster-corrected permutation tests (cluster forming threshold *P* < 0.05).^37^

### Decoding analysis

Data from all trials from each subject were fit using support vector classification decoding analysis using 5-fold cross-validation. In addition, in each fold, each feature in the training set was *z*-scored, and the mean and standard deviation for each feature was used to transform the data in the test set. To handle class imbalances (e.g. more correct versus incorrect trials), models were trained using class weights that were inversely proportional to the class frequency in the training set. This procedure adds a misclassification penalty to the minority class, thus reducing the models bias for the majority class. Performance of the decoding models was assessed using the AUC on the cross-validated predictions. To assess the importance of each feature to the overall performance of the models, feature importance scores were determined using permutation importance, in which each feature was permuted 100 times (while keeping all other features unchanged) and the change in model performance (model loss: 1 − AUC) was computed across all permutations. Because the TLE patient group was older than the HC group, we also performed a separate analysis comparing an age-matched HC versus TLE patient cohort.

## Results

### Subjects

We recruited 14 HCs and 30 TLE patients (Left TLE = 21; Right TLE = 9) (Table 1). Subjects were matched in gender, handedness, MOCA scores, and educational level, but HCs were generally younger (25.4 years) compared to TLE patients (32.1 years, Wilcoxon’s rank sum test, *z* = 2.83, *P* = 0.005, *N* = 44). Descriptive statistics for Left and Right TLE patients demonstrated no difference in demographic features between Left and Right TLE groups; thus, all analyses were performed on all TLE patients as a group (Supplementary Table 1). Individual subject demographics and clinical attributes, including anti-seizure medications, are included in Supplementary Table 2.

**Table 1:** Subject demographics

	HC (*n* = 14)	TLE (*n* = 30)	
Age, mean (SD)	25 (4)	32 (10)	*P* = 0.004*
Gender (female)	7	14	*P* = 0.687
Handedness (RH)	14	26	*P* = 0.1685
MOCA (mean)	28.3	27.1	*P* = 0.0740
Education level			*P* = 0.349
High school	0	0	
Some college	2	6	
College	7	10	
Graduate school	4	10	

### Association performance is worse in TLE patients compared to HCs

**Figure 3:**
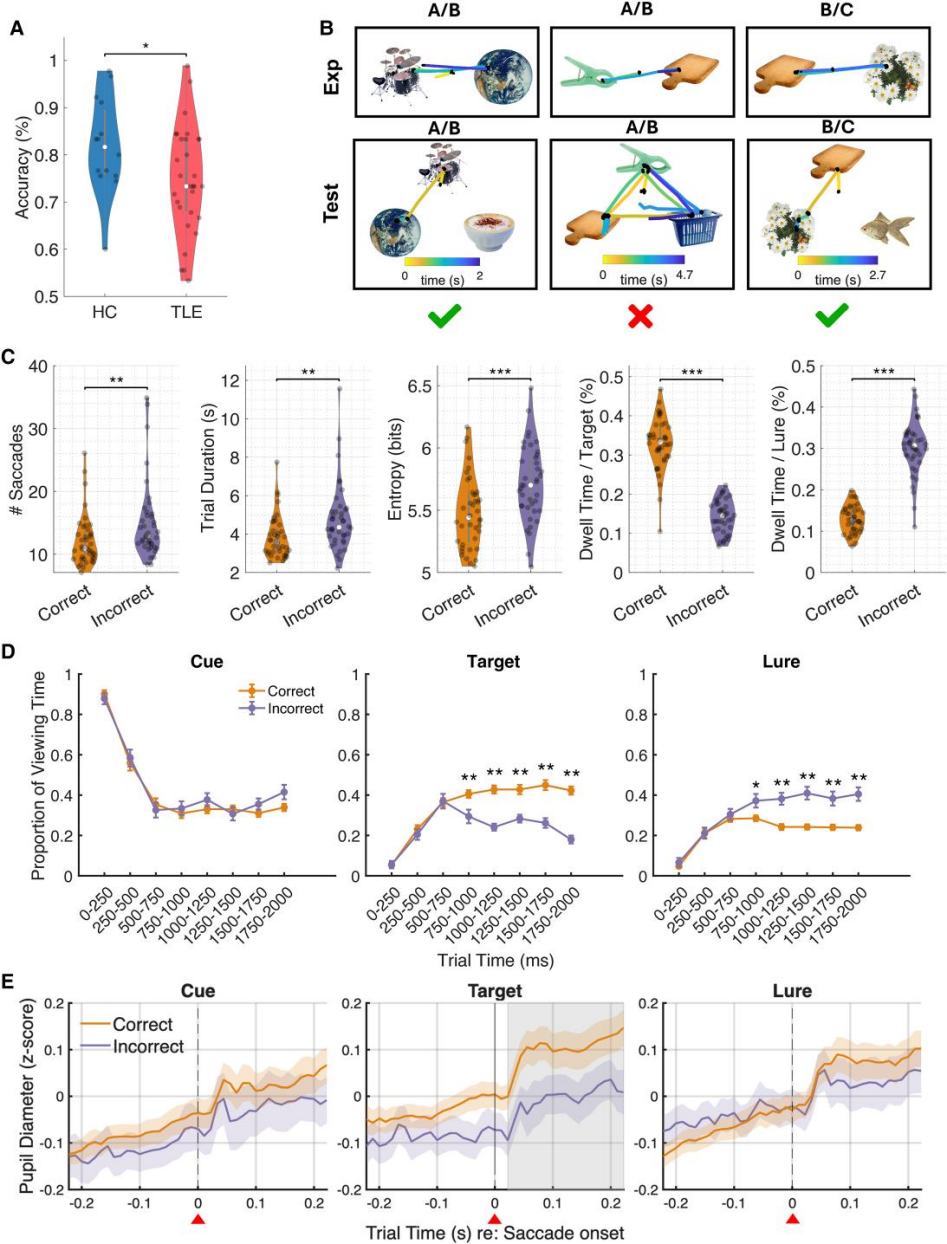
**Eye-movement behaviour tracks with accuracy.** (**A**) TLE patients have modestly decreased accuracy compared to HCs (direct + indirect trials; 75 versus 82%, *P* = 0.031, *z* = 2.16, *N* = 44, Wilcoxon’s rank sum test). Each dot in the violin plot represents the accuracy for an individual subject. (**B**) Example eye-tracking behaviour encoding trials (A/B, B/C) lasting for 1 s, and during correct retrieval trials (green check mark, A/B, A/C) and incorrect retrieval trials (red cross, B/C). (**C**) Correct retrieval trials are characterized by fewer saccades (Wilcoxon’s rank sum test, *z* = −2.80, *P* = 0.005, *N* = 44), shorter trial duration (Wilcoxon’s rank sum test, *z* = −3.28, *P* = 0.001, *N* = 44), lower entropy (Wilcoxon’s rank sum test, *z* = −3.34, *P* = 0.001, *N* = 44), and more dwell time target (Wilcoxon’s rank sum test, *z* = 7.32, *P* < 0.001, *N* = 44) and less time spent on the lure (Wilcoxon’s rank sum test, *z* = −7.35, *P* < 0.001, *N* = 44) when compared to incorrect trials. Each dot in the violin plot represents the accuracy for an individual subject. (**D**) During correct trials, a visual preference for the target is seen at about 750–1000 ms after the test trial starts (Wilcoxon’s rank sum test, *P < 0.05, **P < 0.01, ***P < 0.001, *N* = 44, false-discovery rate-corrected). Error bars represent the mean ± standard error across subjects in each time bin. (**E**) All saccades to cue, target and lure (time = 0, red triangle) are followed by a transient pupillary constriction, followed by a pupillary dilation. However, there is a greater increase in pupil size after saccade to the target object during correct trials (orange) versus incorrect (purple) trials (cluster-corrected permutation test, *P* < 0.05, *N* = 44). There is no difference in pupil size between correct and incorrect trials with saccade to the cue or lure.

There were 2325 exposure + 3367 test trials of usable data collected pooled across subjects. As expected, TLE patients attained a lower accuracy (mean 75 ± 11%) than HCs (mean 82 ± 10%, Wilcoxon’s rank sum test, *z* = 2.08, *P* = 0.019, *N* = 44, one-tailed) across all retrieval trials (direct & indirect). Both groups had above-chance level performance, with worse performance during indirect trials (Fig. 3 and Supplementary Fig. 3). The difference in accuracy was seen mainly in the retrieval of direct associations, with HCs performing at 90 ± 7% accuracy and TLE patients performing at 83 ± 11% accuracy (Wilcoxon’s rank sum test, *z* = 1.83, *P* = 0.034, *N* = 44, one-tailed). Performance on indirect trials was lower than for direct trials, with a non-significant difference in performance between HCs and TLE patients (Wilcoxon’s rank sum, *z* = 1.15, *P* = 0.123, *N* = 44, one-tailed). There was a strong positive correlation between performance accuracy and performance on the MOCA for all subjects (Spearman’s rho = 0.53, *P* < 0.0001, *N* = 39), which was driven mainly by the patient cohort (rho = 0.64, *P* < 0.0001, *N* = 26) (Supplementary Fig. 1A), and true for both direct and indirect trials (Supplementary Fig. 1B).

### Visual scanning patterns differentiate between correct and incorrect trials

Exposure trials were uniformly one second in duration, while the average retrieval trial duration was 3.98 ± 2.61 s with an average inter-saccade interval of 0.26 ± 0.1 s. We next determined which eye-movement patterns characterized accurate versus inaccurate trials (Fig. 3). There was no difference in saccade frequency during the 1-s encoding period of object pairs that were later remembered versus forgotten. There was no difference in saccade velocity during encoding between correct and incorrect trials (Supplementary Fig. 2A), suggesting that attention and motor speed variability did not contribute to poor memory retrieval. However, in the test phase when subjects had unlimited response time, correct retrievals were characterized by fewer saccades (or fixations), shorter trial duration (correct trials: 3.83 ± 1.12 s, incorrect trials: 4.79 ± 1.75 s) and more organized (less chaotic) viewing patterns (Fig. 3B and C). Accurate search patterns were distinguished by more time spent looking at the target compared to the lure (Fig. 3B and C), a pattern that emerged at ∼750–1000 ms after the test trial started. Conversely, incorrect trials were longer and more disorganized, with subjects spending more time on the lure than the target object (Fig. 3B and C). Importantly, there were no significant differences in the average speed of eye movements or time to the first fixation between correct and incorrect trials (Supplementary Fig. 2B). These characteristics were true for both direct and indirect associative learning trials (Supplementary Fig. 3). As such, both sets of trials were pooled together for subsequent analyses.

### Pupil size fluctuations correlate with accuracy

We found saccades to the target object were followed by a transient constriction^38^ and then dilation. The magnitude of pupil dilation was greater during correct versus incorrect trials and was sustained ∼200 ms (Fig. 3E). This difference in pupil size was not seen after saccade to the cue or lure (Fig. 3E).

### Visual scanning patterns differentiate HCs from TLE patients, even for correct trials

**Figure 4:**
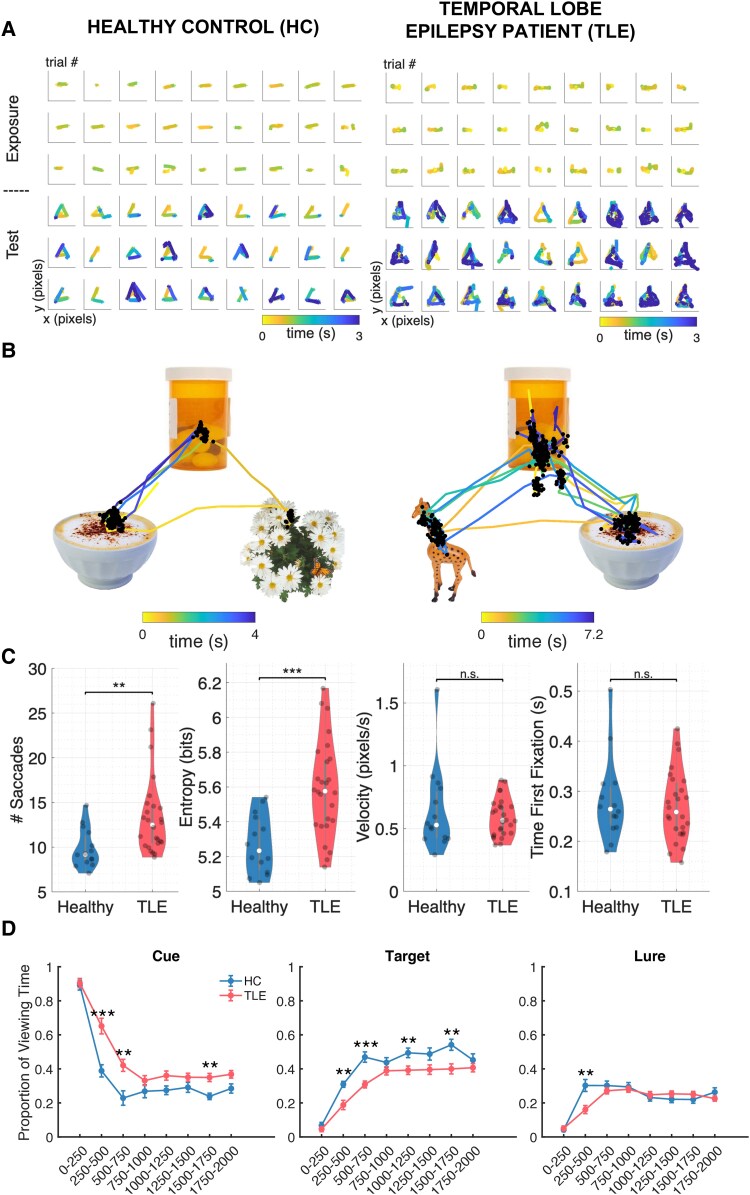
**Saccade behaviour in HCs versus TLE patients during correct trials.** (**A**) Example encoding and retrieval gaze maps for HC and TLE patient qualitatively demonstrate that encoding behaviour is similar, but retrieval scanning differs between HCs and TLE patient, even during correct trials. (**B**) Example eye tracking of HC and TLE patients during a correct association between the pill bottle (cue) and the latte bowl (target). (**C**) TLE patients (red) have more fixations (Wilcoxon’s rank sum test, *z* = 3.11, *P* = 0.002, *N* = 44) and greater entropy (chaos) Wilcoxon’s rank sum test, *z* = 3.02, *P* = 0.003, *N* = 44) during visual retrieval compared to HCs (blue). Importantly, velocity measurements were similar. (**D**) Gaze preference for target over lure within the first 250–500 ms differentiates HCs (blue) from TLE patients (red) (Wilcoxon’s rank sum test, *P < 0.05, **P < 0.01, ***P < 0.001, false-discovery rate-corrected). Error bars represent the standard error across conditions in each time bin.

We next examined whether search patterns can distinguish between HCs and TLE patients. For correct trials, TLE patients had similar eye-tracking behaviour during the 1-s encoding compared to HCs, including number of saccades, entropy and velocity (Fig. 4A). However, during the test phase, HC and TLE behaviour differed, even for correctly retrieved objects during direct and indirect associations (Fig. 4A and B). During the open-ended retrieval period, TLE patients demonstrated a greater number of saccades compared to HCs even on correct trials [13.38 (4.40) versus 9.86 (2.19) saccades, *P* = 0.002, *z* = −3.11], a longer time to first fixation on the target object [1.12 s (0.62) versus (0.76 s (0.1) versus, *P* ≤ 0.001, *z* = −3.63] and more chaotic scanning behaviour [entropy 5.49 (0.26) versus 5.23 (0.15), *P* = 0.003, *z* = −3.02] (Fig. 4C). When we examined eye-movement patterns in a time-resolved manner, we found that TLE patients spent more time on the cue and less time on the target during the first 500 ms of the trial compared to HCs (Fig. 4D). Our results suggest that even when the correct answer is chosen, subtle differences in scan path behaviour between TLE patients and HC can be seen. TLE patients’ scanning behaviour resembles behaviour seen during incorrect trials and implies a weakened or absent memory trace in TLE patients. Visual scanning patterns also suggest that TLE patients, who often have some degree of hippocampal dysfunction, exhibit a range of memory performance (Supplementary Fig. 4A and B). These results imply that eye tracking metrics can decode differences in memory function beyond simple accuracy metrics, by examining the dynamic behavior of eye movements within a trial.

Importantly, patient saccade frequency and velocity during encoding and retrieval were comparable to controls (Fig. 4C), suggesting that differences in motor speed could not explain differences in visual scanning. Many older anti-seizure medications may induce side-effects like blurry and double vision, nystagmus and dizziness. These medications include benzodiazepines, carbamazepine, oxcarbazepine, phenytoin, topiramate, lamotrigine, levetiracetam and gabapentin.^39^ While many of our patients were taking one or more of these medications (Supplementary Table 2), this did not seem to affect their scanning velocity.

### Decoding accuracy and diagnosis by eye-tracking behaviour

**Figure 5:**
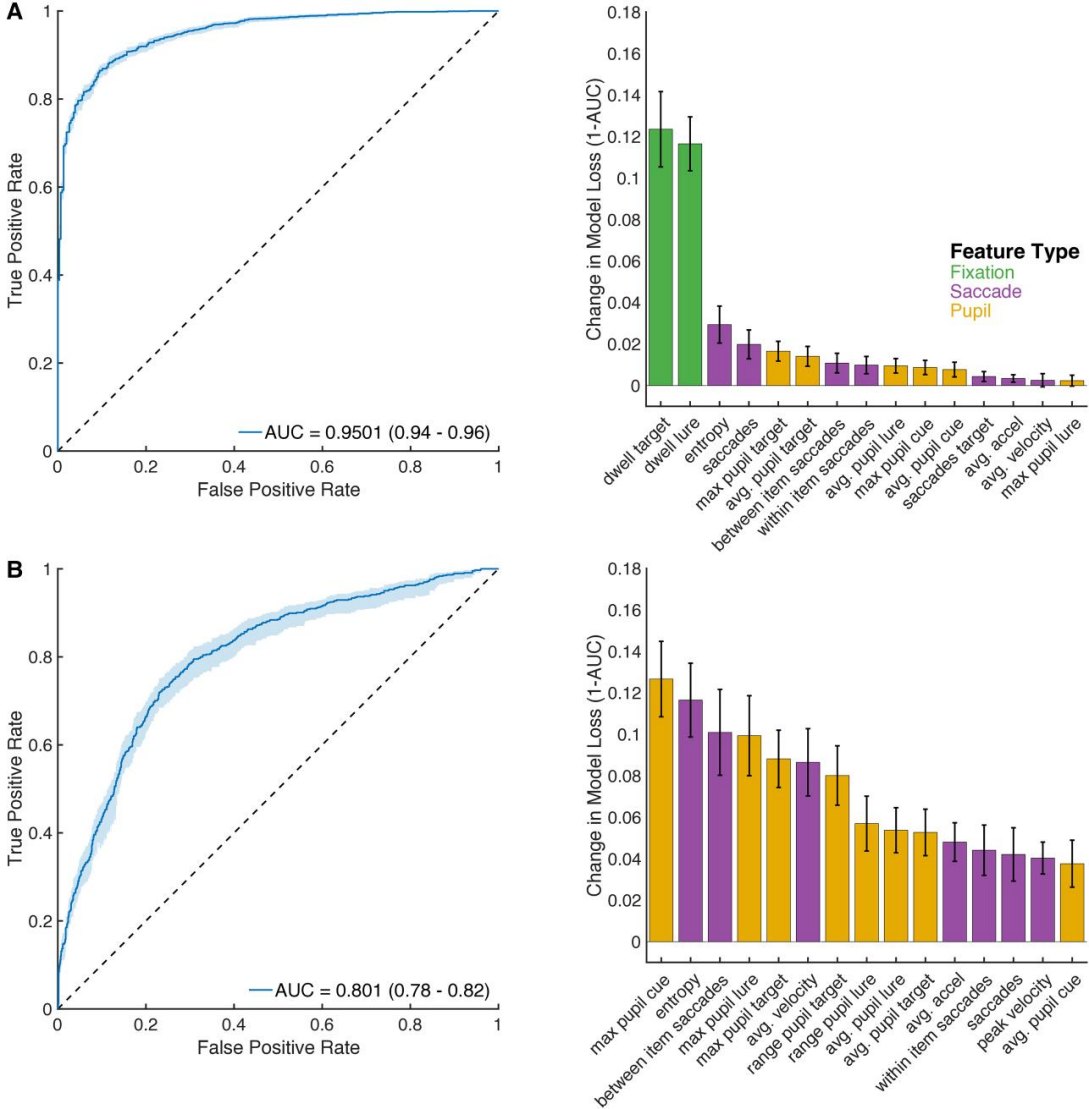
**Decoding performance accuracy and diagnosis from eye tracking features.** (**A**) Left: Receiver Operating Characteristic (ROC) curve (left) for accuracy decoding using all retrieval trials. Error distribution is represented in light blue and represents 95% confidence intervals in ROC. AUC and 95% confidence intervals are reported in the legend. Right: Feature importance scores for the top 15 predictors in this model. Bars represent the mean ( ± standard deviation) in importance score (1 − AUC) for each predictor across 100 permutations of each feature in each cross-validation fold. Green bars signify fixation-related features, Purple bars signify saccade-related features, and Orange bars signify pupil-related features. See Supplementary Table 1 for a full list of features and feature descriptions (avg. = average). (**B**) Left: ROC curve for diagnosis decoding model (HC versus TLE) and right: feature importance scores for the top 15 predictors. Bar colours represent the eye-tracking feature type as in **A**.

Given the quantitative differences we observed in eye-tracking behaviour between correct versus incorrect trials, and HCs versus TLE patients, we built a decoding model for accuracy and diagnosis and determined which eye movement features were most important in classification (Fig. 5). This analysis was restricted to only direct trials as we found a significant difference in overall trial duration between direct and indirect trials (*z* = −11.85, *P* < 0.001, Wilcoxon’s rank sum test, Supplementary Fig. 5) which could potentially bias the models. We found that retrieval performance (correct versus incorrect) could be determined on a per-trial basis with outstanding accuracy (AUC 0.95, Fig. 5A). The most important features for discrimination included relative dwell times on target and lure as well as entropy, contributing to a 25% change in model performance (see Supplementary Fig. 6 for all predictor importance scores). Given that incorrect retrievals were longer in duration, we next limited the analysis to varying time windows, from 1 to 5 s (Supplementary Fig. 7A). As expected, when analysis is limited to the first second of retrieval, decoding performance drops (AUC 0.567). However, with 3 s of data, the model achieved an AUC of 0.868. Four seconds of analysis further improved AUC to 0.9089. To further distinguish the impact of trial duration from other eye-tracking features in predicting accuracy, we ran a decoding model using trial duration with the only feature to achieve an AUC of 0.727 (Supplementary Fig. 7B). Furthermore, feature importance was determined using feature permutation importance. Features such as the amount of time spent looking at the target versus the lure, pupillary dilation when looking at the target, number of between-object saccades and entropy were more important than trial duration (see Supplementary Fig. 6A).

Finally, our model achieved good performance in distinguishing subject diagnosis, even when restricted to correct trials only (Fig. 5B, AUC 0.804). Top predictive features of diagnosis included pupil diameter when viewing the cue, scan path entropy and the number of between-item saccades. Finally, to account for the difference in ages in HCs and TLE patients, we tested the decoding model in age-matched cohorts of HCs and TLE patients. We demonstrated that model performance was stable (AUC 0.8017, Supplementary Fig. 7C). In addition, these models were able to successfully decode between TLE subjects with high versus low MOCA scores (AUC 0.85, Supplementary Fig. 7D), suggesting that eye-tracking features correlate with standard clinical assessments, but are not limited by ceiling effects.

## Discussion

In conclusion, our primary finding is that eye-tracking measures can provide a precise and sensitive measurement of visual association memory compared to explicit measures, such as accuracy or MOCA scores. We demonstrate how features ‘that can only be detected by eye tracking’ can provide useful information that predicts memory accuracy at the single trial level with outstanding performance. In particular, optimal memory performance is characterized by efficient retrieval -- with fewer saccades, early preference for the associated target and a more organized search pattern. Beyond accuracy measurements, eye tracking can reveal the strength of a memory by looking at within-trial dynamics. We have previously shown that the hippocampus supports high-precision features of short-term visual memory, and patients with hippocampal damage make precision errors in visual–spatial associations rather than increases in memory failures *per se*.^40^ This work suggests that eye tracking could be the behavioural correlate of memory precision and strength.

Our secondary finding is that eye tracking features can predict whether a subject is a HC or TLE patient at the single trial level, even among correct trials. As a group, TLE patients have memory impairment compared to HCs due to common but variable hippocampal dysfunction. Thus, even during correct trials, TLE patients on average display scanning features suggestive of a weaker memory trace, such as greater entropy, different pupillary changes to target and lure objects and more saccades between objects. Yet, we also discovered that TLE patients demonstrate heterogeneous visual scanning patterns consistent with a wide range of memory performance, as predicted by their known cognitive phenotypes: (i) normal, (ii) memory and language impaired and (iii) multidomain or globally impaired.^41,42^

### Efficiency of visual search predicts associative memory performance within and between retrieval trials

Saccade patterns could predict accuracy of a single trial with excellent performance (AUC 0.95). Furthermore, eye tracking and pupillometry could decipher mnemonic processes in real time. In all subjects, correct retrieval trials were associated with fewer saccades between objects, leading to a shorter trial duration. Correct trials were distinguished by a more organized search pattern with early preference for the target object with pupillary enlargement on fixation. Conversely, incorrect trials were characterized by a more chaotic search process, with less distinction between target and lure, a longer trial duration and less pupillary discrimination. These findings also align with a prior report of single units in the medial temporal lobe supporting goal-driven identification of a target object during visual search.^43^ During retrieval, target recognition was accompanied by pupillary enlargement on fixation, consistent with previous findings^44,45^ We chose a task that captures an essential function of the hippocampus to associate information across time and space.^46,47^ Patients with hippocampal damage have impaired relational memory.^11,19^ In our tasks, successful retrieval was characterized by more frequent looking between the cue and the target, with an early preference for the target over the lure object. Previous studies that examined faces paired with scenes have also reported more frequent looking between correctly paired faces and scenes than randomly paired faces and scenes, which correlate with bilateral hippocampal fMRI blood-oxygen-level dependent signal, even when explicit memory fails.^2,11^ Our work extends previous visual preference for novelty and relational memory for objects with hippocampal damage, showing that novelty preference worsens with longer delays.^14,24,25,48-50^

On a single trial level, saccades could predict correct association. Prior reports of mnemonically driven visual search reveal that saccade activity seen during the first exposure can be reinstated in the second exposure, but with greater efficiency. Second exposures result in faster search with fewer regressions, or glances backward.^51^  ^,52^ Conversely, repeated searches that too closely resemble the scan path during initial encoding suggest impaired memory function.^53^

Prior literature has established that recall speed is inversely related to object familiarity^54^ and strength of memory (remember/know).^55^ We also found a clear relationship between trial duration and response accuracy. However, we discovered many other features of visual scanning behaviour that predicted trial performance, including relative time spent looking at the target versus the lure, pupillary dilation when looking at the target, number of between-object saccades and entropy. These features were more important than simple measures of response time, which did not contribute significantly to model performance. Importantly, key features of fixation, saccade and pupillary changes can only be detected with an eye tracker.

### Scanning behaviour during a visual association task distinguishes TLE patients from HCs

While there was a modest difference in accuracy seen between TLE patients and HCs (0.73 versus 0.82), eye-tracking metrics differed greatly between subject groups. Tracking patterns could distinguish between HC versus TLE patients on a single trial level with very good performance (AUC 0.80). HCs and TLE patients did not demonstrate any differences in scanning behaviour during encoding (saccades to both objects at similar speeds with a similar number of fixations); however, their retrieval behaviour differed. Even during correct trials, TLE patients had a larger number of saccades and greater entropy (chaos). Additionally, on correct trials, TLE patients demonstrated eye scanning patterns that resembled the disorganized scanning patterns of incorrect trials, suggesting that their answer was weakly certain or even a guess. These behavioural differences could not be attributed to motor or processing speed differences due to epilepsy or medication effect, given that velocity of eye movements was similar between groups. Because all objects that were presented in the retrieval portion were previously shown during encoding, looking behaviour could not be attributed to novelty preference. Overall, TLE patterns of visual scanning during correct trials imply an uncertain or weakened memory trace.

We note that TLE patients were older than HCs, by an average of 7 years. Previous studies have shown that much larger age differences (85 versus 25 years old) account for slower saccade velocity and amplitude, mean and peak velocity.^56^ Because we re-ran the decoding model on age-matched HC and TLE cohorts and found that model performance was stable at predicting diagnosis, older age is highly unlikely to explain group-level differences in scanning behaviour.

Previous reports of animals and humans with medial temporal lobe damage or patients with mild cognitive impairment have reported impaired novelty preference, which becomes greater with longer delays^14,24,25,48-50^. Visual memory tasks involve studying a visual scene, then manipulating part of the scene to see if the subject can recognize the manipulation, or comparison of a novel to familiar object pair. Our findings demonstrate that eye tracking can follow associative memory processes, demonstrated by efficiency of scanning and early preference for the correctly associated object.

Our findings support current understanding of the cognitive phenotype of TLE, with impaired delayed recall.^57^ The specific kind of memory impairment seen in TLE patients has been frequently described as ‘accelerated forgetting’.^58-61^ Accelerated forgetting differs from the cognitive phenotype seen in early Alzheimer’s disease, with both impaired encoding and retrieval, both of which have been attributed to early pathological involvement of the entorhinal cortex.^10^ Both TLE and Alzheimer’s disease patients report ‘subjective’ memory decline that is missed by standardized testing formats, that can predate clinical presentation by years.^5^ Tools such as eye tracking could measure more subtle declines in memory at early disease states and track intra-individual changes over time.

### Pupil size increases after saccade to target object and remains enlarged during fixation during correct trials

Pupillary constriction and dilation are midbrain reflexes that respond to low-level features such as luminance and visual complexity.^62-68^ These reflexes are also modulated by top-down attentional, neuromodulatory and cognitive demands^45,69^ arising from the frontal and parietal regions. Because we compared pupil responses to the same target object by accuracy, we controlled for low-level features that may affect pupil size. Notably, after a brief pupillary constriction associated with the saccade,^38^ pupil sizes increased during the fixation period on all objects (cue, target, lure). However, there was greater pupillary enlargement with saccade to the target object during correct trials results. These findings suggest a top-down neuromodulatory signal at play Pupillary changes to the target and cue are important predictors of trial accuracy (Fig. 5A) and patient diagnosis (Fig. 5B). Overall, this pupillary signal during saccades to the target object is consistent with an early visual preference for the target object. These findings suggest that saccades and pupillary changes occurring early in the trial can predict memory performance much earlier than the explicit response.

Greater pupil size with saccades to target objects during successfully remembered trials is consistent with prior findings of increased pupil size with repeated or familiar objects^70^ or salience of visual, auditory, or visuo-auditory stimuli.^71^ In memory tasks, pupil size has been linked to successful encoding and retrieval of novel word presentations,^44^ with enhanced pupil dilation associated with successful encoding during word-list learning, as well as with successful retrieval of words.^44^ Pupil dilation appears to be especially enhanced for mnemonically driven processes, not solely identification of a target in visual search.^45^

### Clinical implementation

Patients with TLE report memory deficits that are missed by standard neuropsychological tests.^5^ In fact, up to 25% of TLE patients have memory complaints without an objective correlate and are considered ‘subjectively memory impaired’. Our proof of principle study demonstrates how eye tracking can provide behavioural measurements that correlate with existing tests of memory performance (e.g. accuracy and MOCA scores), yet provide much more sensitive measurements that can detect weakened memory trace. Eye tracking avoids the ceiling and floor effects of the MOCA test.

While previous work in memory-guided visual search has focused on group-level effects (e.g, HCs versus mild cognitive impairment, young versus old) or studied patients with hippocampal injury, our findings suggest that eye movements can reveal memory strength in a more nuanced, time-resolved manner. Oculomotor behaviour and pupil size can be used to decipher mnemonic processes at multiple temporal scales, including within and between trials, and predict performance much earlier than the explicit response. Furthermore, tracking features correlate with other external markers of memory function, including accuracy and MOCA scores, but with greater sensitivity.

Thus, eye tracking could be deployed as a complementary assessment to traditional tests and may specifically be used to detect subtle declines over time. Our study was conducted with a portable eye tracker placed at the bottom of a laptop screen. While we used a research grade tracker that had a sampling speed of 120 Hz, a gaming-grade tracker with a frequency of 90 Hz would be sufficient to detect relevant low-frequency saccade patterns and changes in pupillary diameter. In addition, several direct-to-consumer glasses could be used to bring testing into clinical settings. After calibration of the subject’s relative position to the screen, eye movement and pupil activity could be recorded during a visual association task and then measured offline for saccade rate, pupil changes, entropy, gaze preference and task duration. Key saccade features could be measured over time to detect within-subject changes. Previously, novelty-based visual preference tasks have been used to diagnose patients with mild Alzheimer’s disease,^72^ although their clinical implementation is uneven. Because eye tracking can be interpreted without language, it could be useful for memory assessment in autistic, language-impaired and non-English-speaking populations.

### Summary

We have illustrated how eye movements can yield continuous, quantitative measures of episodic memory behaviour, extending simple accuracy or duration metrics used in clinical neuropsychological and cognitive neuroscience.^3^ We have demonstrated that eye tracking can yield memory metrics that are (i) precise (predict trial accuracy with an outstanding performance of 0.95) and correlate with MOCA scores; (ii) sensitive (distinguish between HCs from TLE patients who often have some degree of memory impairment) with very good performance of 0.80 and discover meaningful differences in performance even within correct trials; and (iii) track memory behaviour in a temporally resolved manner. Furthermore, because eye tracking bypasses the need for explicit responses, memory can be disambiguated from speech and language. These features could be useful for testing children, autistic or non-English-speaking populations, as well as patients with early neurodegenerative disease who may have multiple cognitive domains affected. Finally, certain psychometric features of eye tracking such as high temporal precision and a large number of trials are useful for deciphering brain-behavior dynamics iin a real-time manner.

## Supplementary Material

fcaf219_Supplementary_Data

## Data Availability

The data that support the findings of this study are available upon reasonable request to the corresponding authors and after ethics approval.
