# Supplementary material for: Saccades track visual associative memory processes with precision and sensitivity
Source: Brain Commun. 2025 Jun 4;7(3):fcaf219. doi: 10.1093/braincomms/fcaf219 (PMC12204191; doi:10.1093/braincomms/fcaf219)
Supplement: fcaf219_Supplementary_Data [file fcaf219_supplementary_data.pdf]

Metric Label	Description	Units/Type
duration	Trial Duration	Seconds
num fixations	Number of Fixations	Integer
time first_fixation	Time to first fixation	Seconds
time_first_target_fixation	Time to first target fixation	Seconds
dwell_target	Relative time spent looking at the target	Percentage
dwell_lure	Relative time spent looking at the lure	Percentage
dwell_cue	Relative time spent looking at the cue	Percentage
num saccades	Number of saccades	Integer
entropy	Statistical measure of gaze entropy	Bits
path_length	Total length of eye-movement path	norm. pixels
Inter. sacc. interval	Average time between saccades	Seconds
sacc. rate	Average saccade rate	Seconds
avg. saccade	Average saccade duration	Seconds
saccades cue	Number of Saccades to Cue	Integer
saccades target	Number of Saccades to Target	Integer
saccades lure	Number of Saccades to Lure	Integer
num within item saccades	Number of within-object saccades	Integer
num between item saccades	Number of between-object saccades	Integer
avg. velocity	Average eye-movement velocity	mm/s
Peak velocity	Peak eye-movement velocity	mm/s
avg. accel	Average eye-movement acceleration	mm/s^2
Peak accel	Peak eye-movement acceleration	mm/s^2
avg. pupil	Average pupil diameter	Millimeters (mm), z-scored
avg. pupil target	Average pupil diameter when viewing target	Millimeters (mm), z-scored
avg. pupil lure	Average pupil diameter when viewing lure	Millimeters (mm), z-scored
avg. pupil cue	Average pupil diameter when viewing cue	Millimeters (mm), z-scored
max pupil target	Maximum pupil diameter when viewing target	Millimeters (mm), z-scored
max pupil lure	Maximum pupil diameter when viewing lure	Millimeters (mm), z-scored
max pupil cue	Maximum pupil diameter when viewing cue	Millimeters (mm), z-scored
range pupil target	Range of pupil diameter when viewing target	Millimeters (mm), z-scored
range pupil lure	Range of pupil diameter when viewing lure	Millimeters (mm), z-scored
range pupil cue	Range of pupil diameter when viewing cue	Millimeters (mm), z-scored

Key
Fixation
Saccades
Pupil

Supplementary Table 1. All eye-tracking metrics used for analysis along with descriptions and units of measurement. Each metric is color-coded whether it refers to a fixation (green), saccade (purple), or pupil (yellow) –related measure of eye-tracking behavior.

Supplementary Table 2.
Clinical and Demographic Information by Diagnosis and Subject

SUBID	Diagnosis	Gender	Age	Years of Education	MOCA	Anti-Seizure Medications
H070E	TLE	Female	44	College	24	clonazepam, epidiolex, lamotrigine, midazolam, topiramate, olanzapine
H082E	TLE	Female	27	Graduate	28	clonazepam, lamotrigine
H083E	TLE	Female	31	Graduate	26	eslicarbazepine, lacosamide, lamotrigine
H088E	TLE	Female	21	Some	30	clobazam, clonazepam, lamotrigine, levetiracetam
H093E	TLE	Male	29	Some	27	lacosamide, levetiracetam, midazolam
H095E	TLE	Female	20	Some	27	lacosamide
H096E	TLE	Male	24	College	27	clobazam, eslicarbazepine, levetiracetam
H104E	TLE	Female	25	College	28	clobazam, diazepam, folic acid, lacosamide
H108E	TLE	Male	31	College	24	oxcarbazepine
H112E	TLE	Male	NC	NC	NC	oxcarbazepine
H113E	TLE	Male	49	NC	NC	cenobamate, clonazepam, lamotrigine
H140E	TLE	Female	23	Graduate	26	lamotrigine, levitiracetam
H141E	TLE	Male	27	Some	25	levitiracetam, midazolam
H145E	TLE	Female	36	College	26	brivaracetam, cenobamate, clonazepam, folic acid, lacosamide, midazolam
H148E	TLE	Male	35	Graduate	28	lamotrigine, midazolam
H150E	TLE	Female	28	College	26	clonazepam, diazepam, folic acid, levitiracetam
H883E	TLE	Female	61	Graduate	NC	cenobamate, clobazam, folic acid
H915E	TLE	Female	23	Some	23	cenobamate, clobazam, folic acid, midazolam, oxcarbazepine
H922E	TLE	Male	26	NC	26	cenobamate, levitiracetam
H924E	TLE	Female	33	Graduate Leve	33	clobazam, folic acid
H925E	TLE	Female	30	College	30	cenobamate, folic acid, lamotrigine
H084E	TLE	Male	30	College	29	lacosamide, levetiracetam, ritalin
H139E	TLE	Male	48	Some	27	
H147E	TLE	Female	27	Graduate	29	lacosamide, lamotrigine, levetiracetam, midazolam
H149E	TLE	Male	26	College	24	clobazam, lamotrigine, levetiracetam, midazolam
H151E	TLE	Female	36	College	27	lacosamide, lamotrigine
H154E	TLE	Female	30	Graduate	28	lacosamide
H155E	TLE	Male	29	Graduate	NC	lacosamide
H901E	TLE	Female	57	Graduate	29	cenobamate, clonazepam, folic acid, gabapentin, lacosamide, lamotrigine, midazolam
H921E	TLE	Male	28	NC	NC	cenobamate, clobazam , lacosamide, lamotrigine, midazolam, zonisamide
H003H	Healthy	Female	18	Some College	29	N/A
H004H	Healthy	Male	29	College	26	N/A
H005H	Healthy	Female	20	College	30	N/A
H007H	Healthy	Male	28	College	26	N/A
H008H	Healthy	Female	21	Some College	29	N/A
H009H	Healthy	Male	33	Graduate Leve	30	N/A
H026H	Healthy	Male	26	College	30	N/A
H029H	Healthy	Male	29	NC	NC	N/A
H031H	Healthy	Female	22	College	26	N/A
H033H	Healthy	Male	23	College	29	N/A
H034H	Healthy	Female	23	College	27	N/A
H035H	Healthy	Female	24	Graduate	30	N/A
H037H	Healthy	Male	26	Graduate	30	N/A
H038H	Healthy	Female	24	Graduate	26	N/A

*NC= not collected

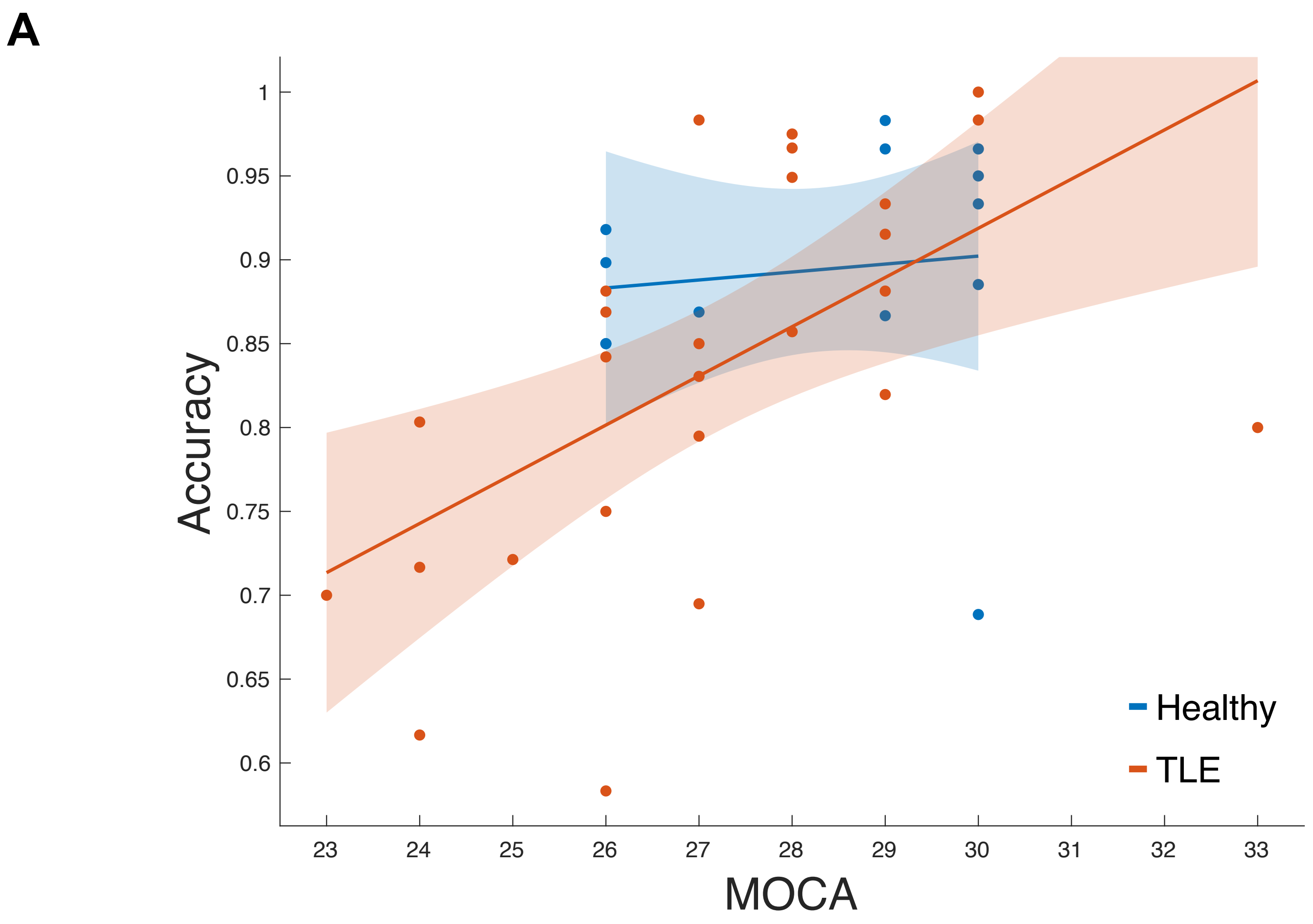

Supplementary Figure 1 A Accuracy is correlated with Montreal Cognitive Assessment (MOCA) scores for all subjects (Spearman's ρ=0.53, p<0.001, N=39), an effect mainly driven by TLE patients (Spearman's ρ=0.64, p<0.001, N=26).

Supplementary Figure 1 (cont.). Accuracy is correlated with MOCA score during both direct (B) and indirect (C) association trials for all subjects (Spearman's ρ_{direct}=0.63, p<0.0001, N=39; $\rho_{indirect}$=0.34, p=0.03), an effect mainly driven by TLE patients (Spearman's ρ_{direct} =0.68, p<0.0001, N=26; $\rho_{indirect}$=0.38, p=0.05).

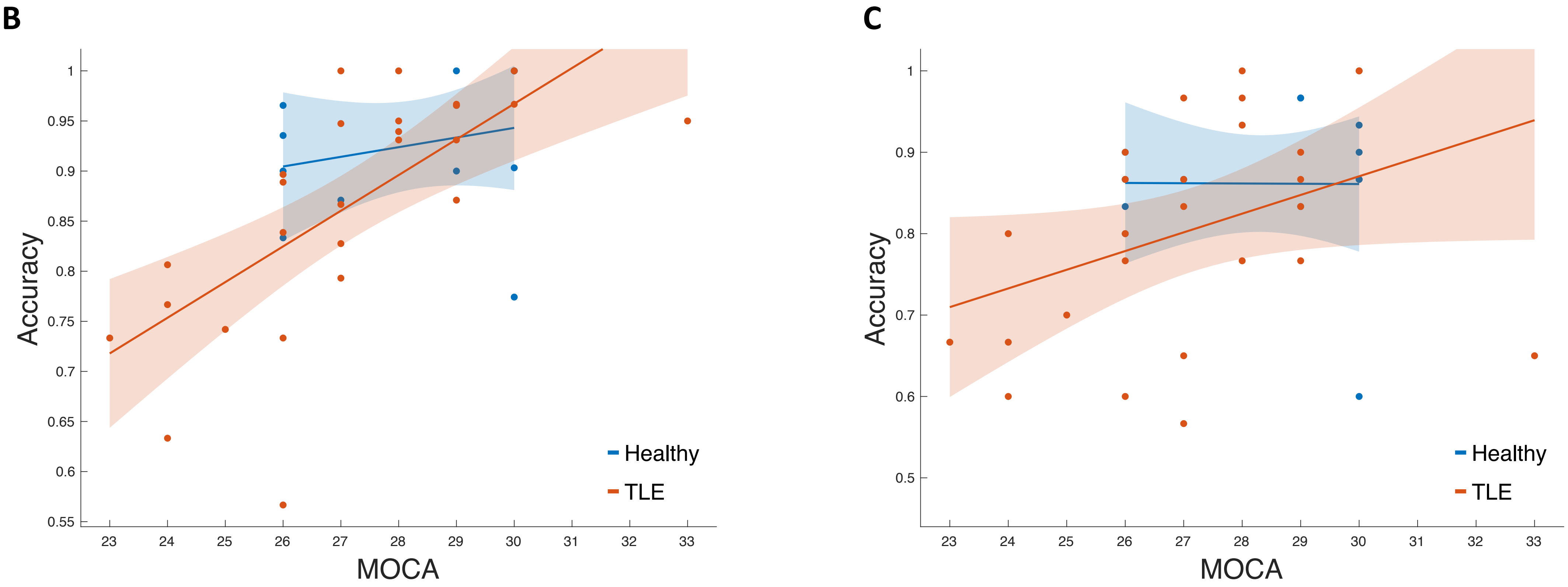

Supplementary Figure 2. **A** No difference between correct and incorrect trials in saccade rate (Wilcoxon's rank sum, z=-0.14, p=0.891, N=44) or velocity (z=0.14, p=0.891, N=44) during encoding. **B** No difference between correct and incorrect trials in saccade rate (z=0.17, p=0.866, N=44) or velocity (z=0.10, p=0.920, N=44) during retrieval. Each dot in the violin plots represents an individual subject average.

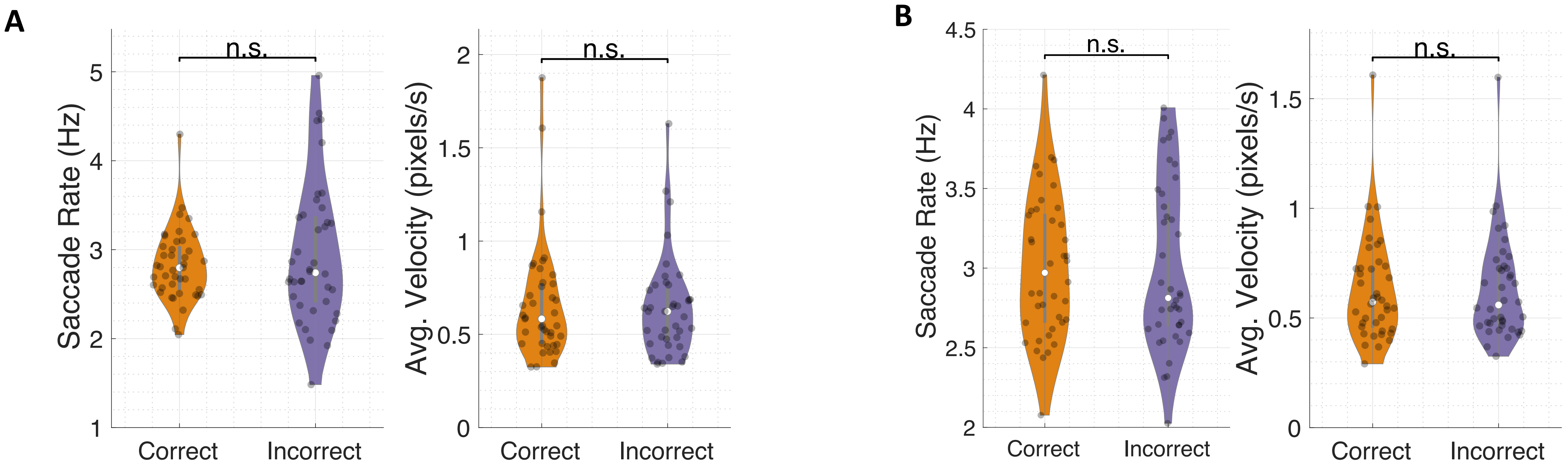

Supplementary Figure 3. Differences between HCs and TLE patients (Wilcoxon rank sum test) in number of saccades (direct: z=-3.33, p=0.001, N=44; indirect: z=-2.45, p=0.014, N=44), trial duration (direct: z=-3.5, p<0.001, N=44; indirect: z=-2.4, p=0.017, N=44) and entropy (direct: z=-3.76, p<0.001, N=44; indirect: z=-2.76, p=0.006, N=44) are seen in direct and indirect trials. No difference was observed for dwell time to target (direct: z=1.40, p=0.16, N=44; indirect: z=0.61, p=0.542, N=44) or dwell time to lure (direct: z=0.69, p=0.487, N=44; indirect: z=0.52, p=0.6, N=44). Each dot in the violin plots represents an individual subject average.

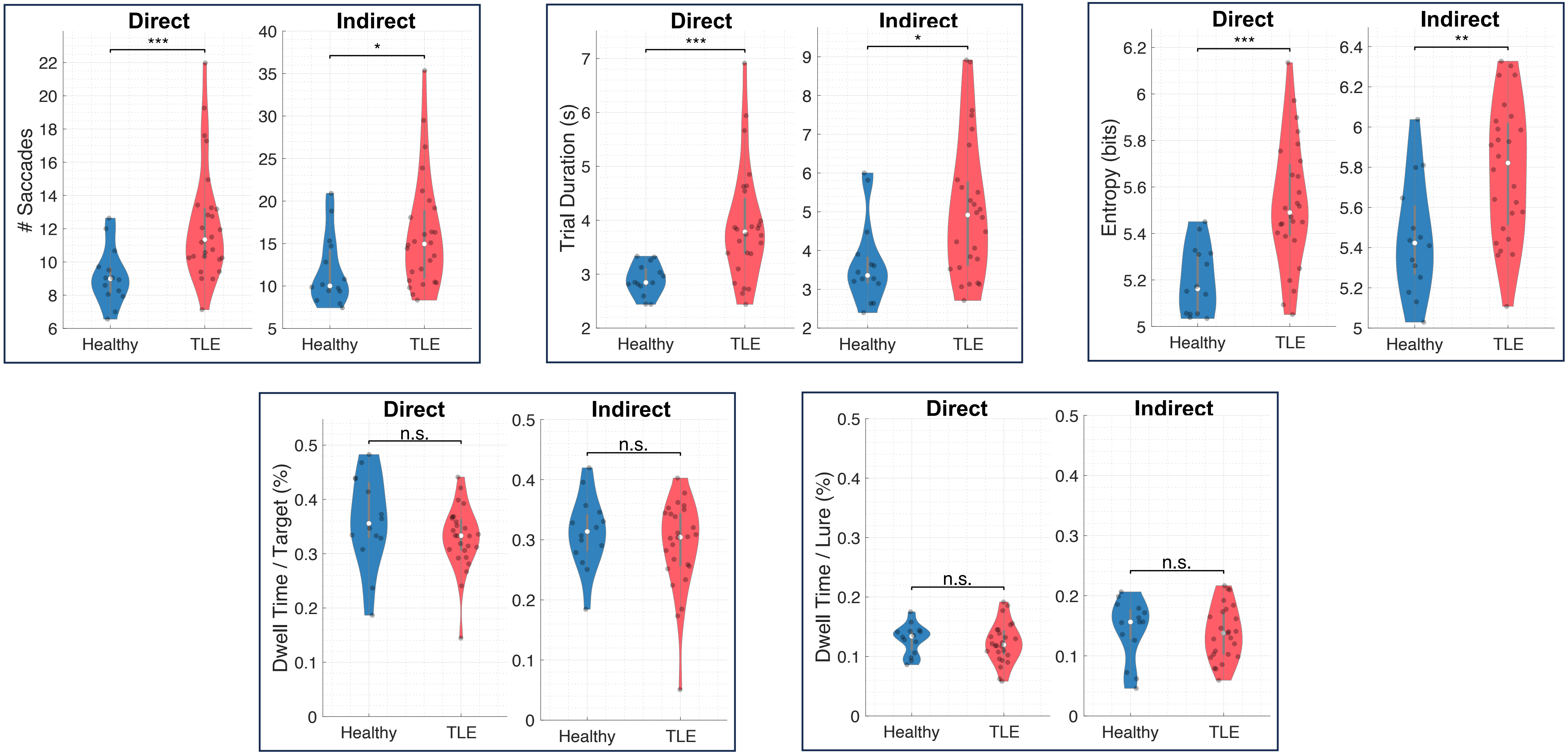

Supplementary Figure 4. A Samples of eye tracking during the exposure (encoding) and test (correct and incorrect retrieval) phases for two randomly chosen healthy patients. **B** Samples of eye tracking during the exposure (encoding) and test (correct and incorrect retrieval) phases for two randomly selected TLE patients.

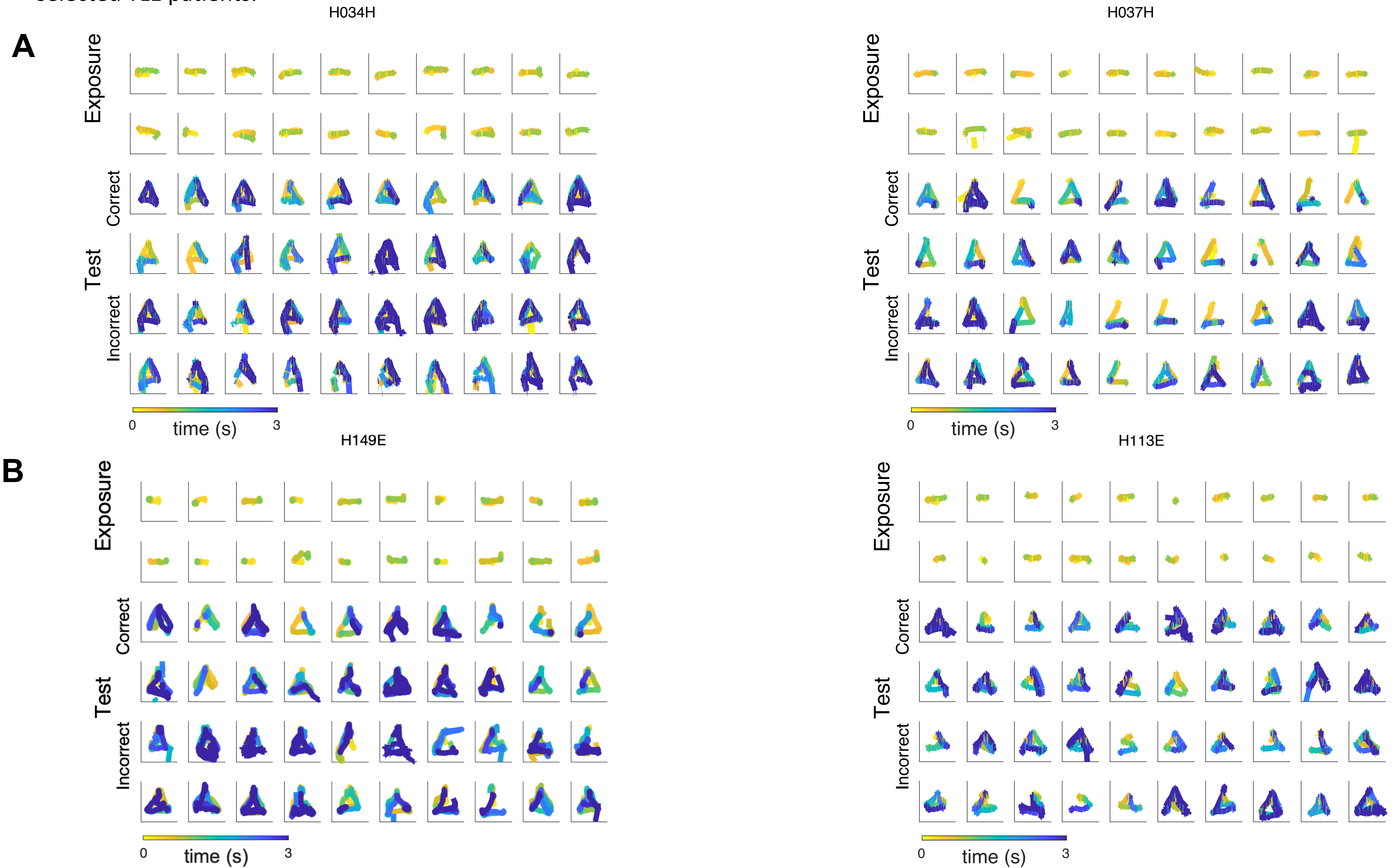

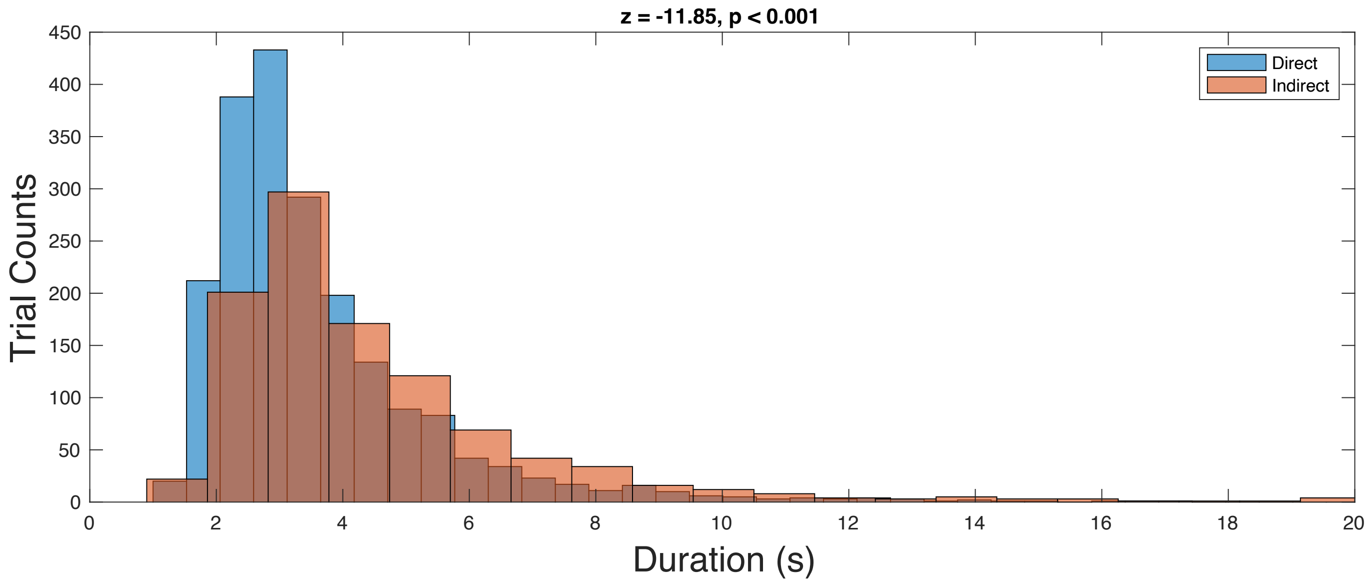

Supplementary Figure 5 – Distribution of trial durations across direct (blue) vs. indirect (red) trials. A significant difference (z=-11.85, $p < 0.001$, Wilcoxon's rank-sum test, N=3367 trials) was observed between the duration of trials in direct vs. indirect conditions.

A Accuracy decoding (all features shown)

B Diagnosis decoding (all features shown)

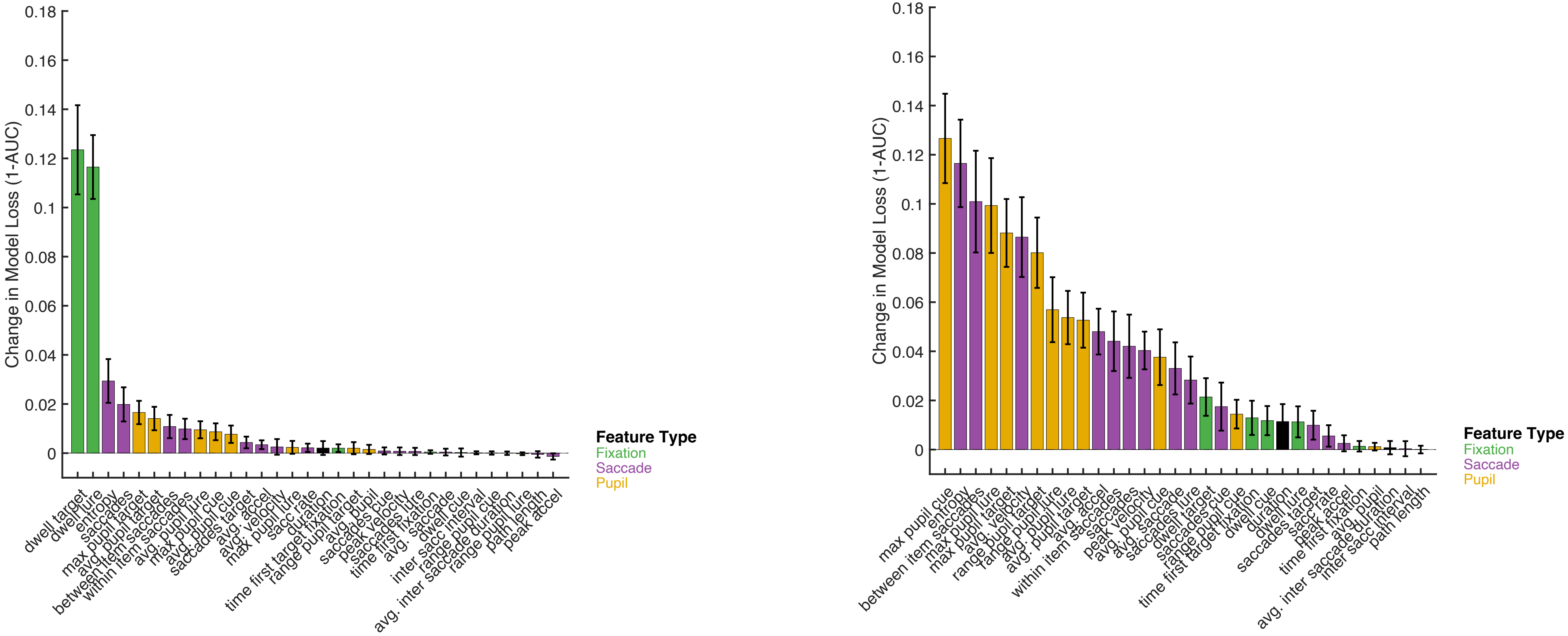

Supplementary Figure 6 – Feature importance scores (1-AUC) for all features used in the decoding models shown in Fig. 5 (A) Scores for all features used in Accuracy decoding model. (B) Scores for all features used in the diagnosis decoding model. Bar colors represent the eye-tracking feature type (Green: Fixation-related, Purple: Saccade-related, Orange: Pupil-related). Bars show the mean importance score and error bars represent the pooled standard deviation in importance scores calculated across 100 permutations of each feature. See Supplementary Table 1 for a full list of features and descriptions.

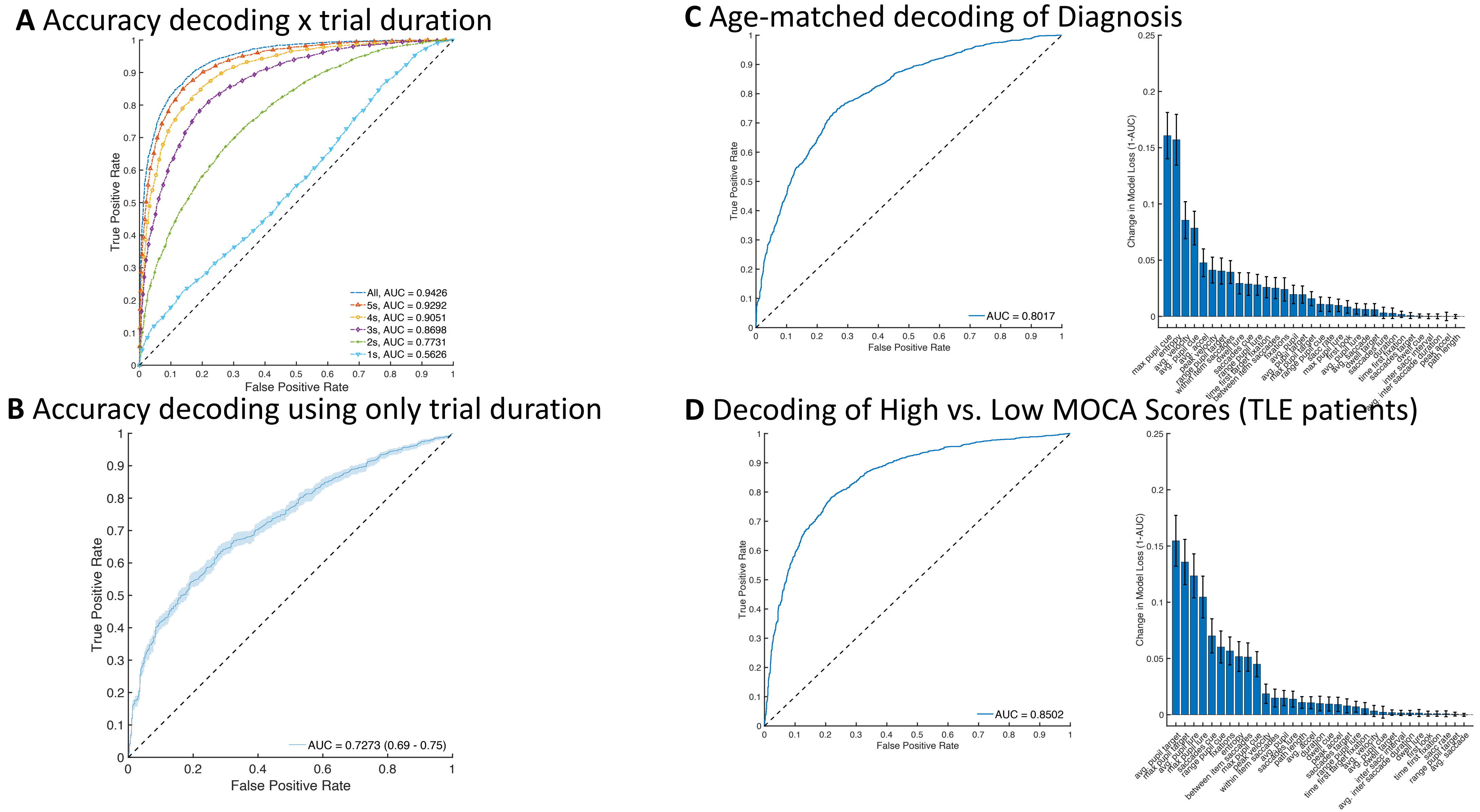

Supplementary Figure 7 – A Accuracy decoding using different trial lengths. For each trial length, data from all trials was truncated up to the time indicated. As more eye-tracking data is truncated (e.g., 5s, 4s, ..1s) decoding accuracy declined. **B** Accuracy decoding using only trial duration as a single predictor showed poor decoding ability (AUC 0.73). **C** Diagnosis decoding using an age-matched subset of HC and TLE subjects. **D** Decoding of individual MOCA category (high-moca [>= 27] vs. low-moca [< 27]). Bar plots show the mean importance score and error bars represent the pooled standard deviation in importance scores calculated across 100 permutations of each feature. See Supplementary Table 1 for a full list of features and descriptions.
